# Soluble mannose receptor CD206 and von Willebrand factor are early biomarkers to identify patients at risk for severe or necrotizing acute pancreatitis

**DOI:** 10.1186/s40560-022-00619-2

**Published:** 2022-06-11

**Authors:** Philipp A. Reuken, Jonathan F. Brozat, Stefanie Quickert, Oluwatomi Ibidapo-obe, Johanna Reißing, Anika Franz, Sven Stengel, Ulf K.-M. Teichgräber, Michael Kiehntopf, Christian Trautwein, Andreas Stallmach, Alexander Koch, Tony Bruns

**Affiliations:** 1 Department of Internal Medicine IV, Jena University Hospital, Friedrich Schiller University Jena, Am Klinikum 1, 07747 Jena, Germany; 2 Department of Internal Medicine III, University Hospital RWTH Aachen, Aachen, Germany; 3 Department of Radiology, Jena University Hospital, Friedrich Schiller University Jena, Jena, Germany; 4 Department of Clinical Chemistry and Laboratory Diagnostics, Jena University Hospital, Jena, Germany

**Keywords:** Acute pancreatitis, Necrotizing pancreatitis, Endothelial activation, Macrophage activation

## Abstract

**Background:**

In acute pancreatitis (AP), microcirculatory dysfunction and leukocyte activation contribute to organ damage, inflammation, and mortality. Given the role of macrophage activation, monocyte recruitment, and microthrombus formation in the early pathogenesis of AP, we examined the macrophage activation marker soluble mannose receptor (sCD206) and the endothelial function marker von Willebrand factor (vWF) in patients admitted for AP.

**Methods:**

In an exploratory analysis, serum sCD206 and plasma vWF were prospectively analyzed on day 1 and day 3 in 81 patients with AP admitted to the hospital. In addition, blood samples from 59 patients with early AP admitted to the intensive care unit and symptom onset < 24 h were retrospectively analyzed. Patients were dichotomized as per study protocol into two groups: (i) “non-severe edematous AP” including patients with mild AP without organ failure and patients with transient organ failure that resolves within 48 h and (ii) “severe/necrotizing AP” including patients with severe AP and persistent organ failure > 48 h and/or patients with local complications.

**Results:**

In the prospective cohort, 17% developed severe/necrotizing pancreatitis compared with 56% in the ICU cohort. Serum concentrations of sCD206 on admission were higher in patients with severe/necrotizing AP than in patients with non-severe edematous AP (prospective: 1.57 vs. 0.66 mg/l, *P* = 0.005; ICU: 1.76 vs. 1.25 mg/l, *P* = 0.006), whereas other inflammatory markers (leukocytes, C-reactive protein, procalcitonin) and disease severity (SOFA, SAPS II, APACHE II) did not show significant differences. Patients with severe/necrotizing AP had a greater increase in sCD206 than patients with non-severe edematous AP at day 3 in the prospective cohort. In contrast to routine coagulation parameters, vWF antigen levels were elevated on admission (prospective cohort: 375 vs. 257%, *P* = 0.02; ICU cohort: 240 vs. 184%, *P* = 0.03). When used as continuous variables, sCD206 and VWF antigen remained predictors of severe/necrotizing AP after adjustment for etiology and age in both cohorts.

**Conclusions:**

sCD206 identifies patients at risk of severe AP at earlier timepoints than routine markers of inflammation and coagulation. Prospective studies are needed to investigate whether incorporating early or repeated measurements into the existing scoring system will better identify patients at increased risk for complications of AP.

**Supplementary Information:**

The online version contains supplementary material available at 10.1186/s40560-022-00619-2.

## Background

Acute pancreatitis (AP) is a frequent and potentially life-threatening disease with an incidence of approximately 111 per 100,000 adults in the United States^1^. In most patients, AP has a mild course in the absence of both local complications and persistent organ failure^2^. However, about 20% of patients develop a severe course of AP, with mortality rates ranging from 15 to 35%^3^. To identify patients at high risk for complications, several scoring systems have been evaluated: the Ranson score^4^, the Acute Physiology and Chronic Health Evaluation (APACHE) II score^5^, and the sequential organ failure assessment (SOFA) score^6^. Whereas their accuracy in predicting persistent organ failure is comparable but modest^7^, scores are often too complex and hard to apply in medical settings outside intensive care units (APACHE-II, SOFA). More convenient scoring systems, such as the bedside index for severity in acute pancreatitis (BISAP)^8^, suffer from low sensitivity for predicting patient mortality^8^. Single routine laboratory parameters such as hematocrit or blood glucose often have poor predictive value for detecting severe courses of AP^9,10^. In addition, sonography for diagnosing pleural effusion (BISAP) is not always readily available.

AP is classically understood as the premature activation of trypsin in pancreatic acinar cells followed by autodigestion, pancreatic inflammation and subsequent systemic inflammatory response^11^. In severe AP, myeloid cells are the predominant immune cells migrating to the pancreas and contribute to organ damage, inflammation, and finally mortality^12–14^. Inflammatory macrophages infiltrate the pancreas early after the injury and upregulate markers of alternative activation within interlobular areas of the inflamed pancreas^15^. Within the first 12 h after AP induction, surface expression of CD206 by pancreatic macrophages is dramatically decreased^14^, which may be due to dilution by infiltrating immune cells, downregulation of surface expression, or shedding from the surface. The shedding of CD206 from macrophages is mediated by proteases in response to activation via Protein C kinase, ATP, ligation of TLR2 or dectin-1 or after infection with *Candida albicans*, *Aspergillus fumigatus, Pneumocystis carinii*, and *E. coli*^16–19^, although the exact underlying mechanisms are not fully known.

As a consequence of pancreatic inflammation, microcirculatory dysfunction occurs early in the course of AP, disturbing capillary blood flow and increasing capillary permeability. Thus, further promoting local and systemic leukocyte activation^20^. A hallmark of inflammatory endothelial injury is the degranulation of endothelial cells and the release of von Willebrand factor (vWF). vWF promotes platelet adhesion and aggregation, stimulating the formation of platelet microthrombi and thereby illustrating the close interaction of inflammatory compounds with the coagulation system^21–23^ in the context of AP. vWF^24–27^ and its cleaving protease, ADAMTS13, a disintegrin and metalloproteinase with a thrombospondin type 1 motif, capable of cleaving vWF multimers into smaller forms^28,29^ have been implicated in the pathogenesis and severity of AP.

Given the key roles of macrophages and microthrombi formation in the pathogenesis of severe AP, this study investigated, whether surrogates of macrophage activation (sCD206) and endothelial dysfunction (vWF) can be employed as early biomarkers, possibly predicting the course of AP. Therefore, we performed a prospective exploratory analysis of sCD206 and plasma vWF in patients hospitalized for AP (prospective cohort) and in patients with AP admitted to the ICU for intensified monitoring or treatment (retrospective ICU cohort).

## Study design and methods

### Study population

Eighty-one consecutive hospitalized patients with AP between May 2017 and October 2018 were prospectively enrolled at the Jena University Hospital. Acute pancreatitis was defined as a combination of two or more of the following symptoms: elevated serum lipase more than threefold, typical abdominal pain or typical imaging findings^2^. Exclusion criteria were concomitant cirrhosis, von-Willebrand syndrome, thrombotic thrombocytopenic purpura and pregnancy. Patients were dichotomized as per study protocol into two groups: (i) “non-severe edematous AP” including patients with mild AP without organ failure and patients with transient organ failure that resolves within 48 h and (ii) “severe/necrotizing AP” including patients with severe AP and persistent organ failure > 48 h and patients with local complications^2^. A dichotomization was performed for considerations of sufficient statistical power as we expected only 5% to 10% patients to develop persistent organ failure^30^. Patients were followed-up for in-hospital death or discharge from the hospital. Demographic and clinical data including age, sex, date of hospitalization, and comorbidities according to Charlson Comorbidity Index^31^ were collected at hospital admission.

An independent retrospective cohort included 59 patients with AP admitted to the medical intensive care unit (ICU) of Aachen University Hospital between 2010 and 2017. Only patients in whom the documented time from symptom onset (pain) to ICU admission and sample collection was 24 h or less were considered for analysis. Patients were dichotomized into patients with severe or necrotizing AP (n = 33) and non-severe edematous AP (n = 26) as described above. Patients were followed-up for in-hospital death or discharge from the hospital.

In both cohorts, the diagnosis of necrotizing pancreatitis was made by an experienced radiologist using computed tomography with intravenous contrast medium according to routine clinical practice. In a few patients in whom computed tomography could not be performed or was contraindicated, the diagnosis of pancreatic necrosis was made by endoscopic ultrasound. With the indication for imaging given by the treating gastroenterologist or intensivist, a total of 41 patients in the prospective group and 41 patients in the ICU cohort underwent computed tomography or endoscopic ultrasound.

All patients, next of kin or legal guardians gave written informed consent according to the study protocols prior to inclusion as approved by the respective internal review boards and ethics committee (5128–03/17, Jena and EK 150/06, Aachen).

### Plasma and serum analysis

Blood samples were obtained at hospital admission and on day 3 in the prospective cohort and at ICU admission in the retrospective ICU cohort. Citrated blood samples were centrifuged at 1800 × g for 15 min to obtain platelet-reduced plasma; serum samples were centrifuged at 1800 × g for 10 min. Samples were aliquoted and stored at -80 °C until measurement.

Soluble CD206 (sCD206) was determined in serum using the human soluble mannose receptor (sMR) ELISA Kit (Hycult Biotech, Uden, Netherlands) according to the manufacturer’s instructions. Citrated plasma was thawed for 10 min in a 37 °C water bath to avoid formation of cryoprecipitates. ADAMTS13 activity and antigen levels were measured in prospective cohort using a commercially available fluorogenic enzyme-linked immune-sorbent assay (ELISA) (Technoclone, Vienna, Austria) according to the manufacturer’s instructions. vWF antigen (vWF:Ag) and vWF ristocetin cofactor activity (vWF:RCo) were determined in both cohorts as described previously^32^.

Total bilirubin, albumin, creatinine, urea, sodium, calcium, international normalized ratio (INR), alanine aminotransferase (ALT), aspartate aminotransferase (AST), alkaline phosphatase (ALP), gamma-glutamyl transferase (GGT), lipase, amylase, glucose, lactate, pH, fibrinogen, D-dimer, C-reactive protein (CRP), procalcitonin (PCT), white blood cell count (WBC), hemoglobin, hematocrit and platelet count were determined using routine laboratory analysis.

Surface expression of HLA-DR and MERTK on circulating monocytes was determined as described in the Additional file 1.

### Statistical analysis

Data are expressed as medians with interquartiles and visualized using box plots with individual data points unless otherwise indicated. Differences between groups were analysed by the Fisher’s exact test, Wilcoxon signed-rank test for paired samples or the Mann–Whitney *U* test or the Kruskal–Wallis test for unpaired samples as appropriate. Diagnostic accuracy to distinguish between severe or necrotizing AP and non-severe edematous AP was assessed using the area under the receiver operating characteristics curve (AUROC). Binary logistic regression analysis was performed to calculate odds ratio (OR) of sCD206, vWF:Ag and vWF:RCo to identify patients with severe/necrotizing AP on day 1 (prospective and ICU cohort) and 3 (prospective cohort only). Multivariable binary logistic regression was performed to adjust for AP etiology and age. Statistical analysis was performed using SPSS v27 (IBM, Armonk, NY, USA) and Prism v8 (GraphPad, La Jolla, CA, USA). A two-sided significance level of p < 0.05 was applied. Systematic randomization, correction for multiple testing and blinding were not performed.

## Results

### Prospective cohort

Out of 81 patients who were admitted to the Jena University Hospital for AP, 14 (17%) developed severe or necrotizing pancreatitis, including 10 with necrotizing AP, two with persistent organ failure, and two with both, necrosis and organ failure. The median time from admission to the diagnosis of necrosis was 8 days (interquartile range, 6 to 9). The median time from admission to the diagnosis of persistent organ failure was 2 days (interquartile range, 1 to 4).

Patients who developed severe/necrotizing AP were more frequently transferred to intensive or intermediate care (64% vs. 15%, *P* = 0.001) and had longer hospital stay (median 20 vs. 8 days, p < 0.001) than patients with non-severe edematous AP (Additional file 1: Table S1). In-hospital mortality in patients with severe/necrotizing AP was 7% as compared to 0% in non-severe edematous AP.

Patients with severe/necrotizing AP were younger than patients with non-severe edematous AP and more often had alcoholic pancreatitis. They presented more often with tachycardia without significant differences in routine laboratory parameters on admission (Table 1). Forty-eight hours after admission (day 3), hematocrit, total serum calcium, albumin, lipase, amylase, urea, lactate dehydrogenase, and D-Dimers were higher in patients with severe/necrotizing AP than in patients with non-severe edematous AP (Table 1).

**Table 1:** Clinical characteristics of patients with acute pancreatitis (Prospective cohort)

	Day 1 (Admission to hospital)			Day 3		
Characteristics	Severe or necrotizing (*n* = 14)	Non-severe edematous (*n* = 67)	*P* value	Severe or necrotizing (*n* = 14)	Non-severe edematous (*n* = 67)	*P* value
Age (years)	48 (41; 59)	69 (52; 78)	0.001			
Male sex	10 (71%)	39 (58%)	0.55			
Body weight (kg)	78 (67.25; 101.25)	83 (71; 92)	0.97			
BMI (kg/m^2^)	28.2 (23.5; 31.1)	27.7 (25.0; 31.0)	0.64			
Etiology						
Biliary Alcoholic Other	2 (14%) 8 (57%) 4 (29%)	31 (46%) 5 (7%) 31 (46%)	0.001			
Alcohol use (n)	12 (86%)	23 (34%)	0.001			
Smoker (n)	5 (36%)	12 (15%)	0.16			
Hemoglobin (mmol/l)	9.0 (7.9; 10.4)	8.2 (7.5; 9.3)	0.18	6.7 (6.4; 7.7)	7.6 (6.9; 8.1)	0.05
Hematocrit (%)	39 (36; 48)	40 (36; 44)	0.92	32 (30; 37)	37 (33; 38)	0.04
INR	1.0 (1.0; 1.4)	1.1 (1.1; 1.3)	0.39	1.2 (1.1; 1.5)	1.2 (1.1; 1.3)	0.68
PTT (sec)	26.8 (24.3; 30.2)	27.6 (35.4; 30.0)	0.36	27.6 (26.0; 29.4)	28.3 (26.9; 30.9)	0.23
Platelets (/nl)	195 (166; 347)	254 (183; 297)	0.61	148 (116; 213)	203 (155; 259)	0.07
Ca^2+^ (mmol/l)	2.21 (1.93; 2.49)	2.39 (2.21; 2.73)	0.07	1.98 (1.70; 2.10)	2.23 (2.15; 2.30)	< 0.001
Ca^2+^ corrected (mmol/l)	2.48 (2.27; 2.68)	2.49 (2.36; 2.60)	1.00	2.50 (2.26; 2.53)	2.49 (2.39; 2.61)	0.52
LDH (µmol/l/s)	4.33 (3.32; 4.74)	4.31 (3.36; 6.22)	0.75	5.45 (4.97; 14.83)	4.02 (3.31; 5.14)	0.002
Bilirubin (µmol/l)	16.5 (9.75; 34.75)	32 (14; 56.25)	0.11	14 (10.25; 22)	18 (12; 31.5)	0.32
ALP (µmol/l/s)	1.33 (1.15; 2.22)	2.04 (1.32; 3.72)	0.06	1.48 (1.00; 2.27)	1.54 (1.06; 3.06)	0.44
GGT (µmol/l/s)	3.10 (1.14; 4.49)	4.99 (1.05; 8.75)	0.46	2.00 (0.80; 6.16)	2.83 (0.86; 7.38)	0.55
Amylase (µmol/l/s)	7.45 (6.17; 18.54)	13.74 (3.96; 28.40)	0.52	6.5 (2.41; 11.53)	1.70 (0.86; 3.20)	0.02
Lipase (µmol/l/s)	31.64 (9.85; 53.45)	43.08 (9.84; 97.75)	0.38	5.60 (3.03; 11.07)	2.06 (1.07; 4.85)	0.004
ALT (µmol/l/s)	0.49 (0.27; 0.88)	1.64 (0.43; 4.40)	0.06	0.29 (0.19; 0.87)	1.10 (0.29; 2.62)	0.03
AST (µmol/l/s)	0.88 (0.48; 1.70)	1.52 (0.53; 3.58)	0.20	0.89 (0.72; 1.48)	0.67 (0.43; 1.19)	0.15
Albumin (g/l)	29 (26; 38)	34 (31; 38)	0.15	21 (17; 24)	29 (26; 32)	< 0.001
pH	7.40 (7.36; 7.44)	7.41 (7.38; 7.44)	0.46	7.39 (7.33; 7.43)	7.40 (7.38; 7.43)	0.38
Lactate (mmol/l)	1.47 (1.03; 2.10)	1.40 (1.05; 2.09)	0.89	0.85 (0.70; 1.10)	1.01 (0.78; 1.25)	0.32
Glucose (mmol/l)	8.4 (5.7; 10.2)	7.0 (5.8; 8.8)	0.26	7.9 (4.4; 9.4)	5.9 (5.1; 7.2)	0.25
Urea (mmol/l)	6.6 (5.9; 10.5)	6.0 (4.2; 8.1)	0.40	6.5 (4.4; 12.0)	4.6 (3.1; 6.8)	0.04
Creatinine (µmol/l)	79 (69.5; 157.5)	77 (68; 102.5)	0.37	85.5 (55.5; 135.75)	69 (61; 88.25)	0.34
Standard bicarbonate (mmol/l)	23.6 (21.5; 26.1)	24.6 (23.1; 26.3)	0.37	21.3 (19.1; 27.4)	25.2 (23.8; 26.7)	0.08
Base Excess (mmol/l)	0.3 (-4.8; 1.4)	0.9 (-1.3; 2.9)	0.13	-2.5 (-6.5; 3.7)	1.1 (-0.4; 2.6)	0.11
SOFA score	1.0 (0.5; 2.5)	2 (0; 2.25)	0.95	1.0 (1.0; 3.75)	1.0 (0; 2)	0.10
BISAP score	1 (0; 1,75)	1 (1; 2)	0.53	N/A		
SAPS II score	17 (13; 24)	22 (16; 26)	0.11	18 (11.5; 30.5)	20 (7; 25)	0.75
APACHE II score	7 (5.75; 8.75)	8 (5; 13)	0.27	N/A		
CCI (points)	1.5 (0; 2)	3 (1; 6)	0.008	N/A		
Ranson Criteria	N/A			2.5 (0.25; 3.75)	1 (0; 2)	0.10
SIRS (points)	1.5 (0.5; 2)	0.5 (0; 1)	0.005	N/A		
CRP (mg/l)	82.5 (8.7; 222.2)	24.9 (3.8; 70.9)	0.12	367 (306; 400)	127 (35; 215)	< 0.001
PCT (ng/ml)	0.48 (0.93; 2.80)	0.22 (0.07; 0.51)	0.49	1.81 (0.34; 7.34)	0.19 (0.11; 0.73)	0.002
WBC (/nl)	14.6 (9.1; 18.3)	11.9 (9.0; 15.9)	0.16	12.3 (8.3, 17.5)	9.7 (7.1; 11.6)	0.046
sCD206 (mg/l)	1.57 (0.59; 1.89)	0.66 (0.51; 0.95)	0.005	2.41 (1.31–2.58)	0.86 (0.59; 1.23)	< 0.001
Fibrinogen (g/l)	3.2 (2.7; 4.7)	3.8 (3.2; 4.7)	0.50	8.3 (3.9; 10.0)	5.0 (3.8; 6.2)	0.06
D-Dimer (µg/l)	896 (312; 1948)	468 (310; 864)	0.26	2833 (1578; 4531)	646 (355; 1169)	< 0.001
vWF:Ag (%)	375 (285; 401)	257 (204; 355)	0.02	393 (288; 580)	235 (198; 342)	0.001
vWF:RCo (%)	401 (302; 433)	266 (194; 339)	0.01	428 (319; 646)	255 (200; 352)	< 0.001

*P* values from Mann–Whitney *U* test or Fisher’s exact test. Medians with first and third quartile or frequencies with percentages are shown. *N/A* not applicable

SOFA, BISAP, Simplified Acute Physiology Score (SAPS) II, APACHE II, and the Ranson criteria were not able to discriminate between patients with severe/necrotizing and non-severe edematous courses of AP at day 1 or day 3 in the prospective cohort (Table 1).

### ICU cohort

Because only 14 (17%) patients in the prospective cohort developed severe or necrotizing pancreatitis, we also included an independent retrospective cohort of patients enriched in more severe courses. Patients were eligible for analysis, if the time from symptom onset to ICU admission and sample collection was 24 h or less. Main reasons for admission to ICU were organ failure, management of alcohol withdrawal symptoms or pain, preparation for endoscopic procedures for impacted bile duct stones or intensified monitoring as outlined in the Additional file 1:Table S2.

In the ICU cohort, 33 (56%) patients developed severe/necrotizing AP and 26 (44%) had non-severe edematous pancreatitis. Twenty patients had organ failure and necrosis, 13 patients had organ failure only. The median time from ICU admission to diagnosis of necrosis by computed tomography or endoscopic ultrasound was 5 days (interquartile range 4 to 7), and the median time from ICU admission to onset of persistent or transient organ failure was 0 days (interquartile range 0 to 1). The in-hospital mortality in patients with severe/necrotizing AP was 4/33 (12%) as compared to 0% in patients with non-severe edematous AP (Additional file 1: Table S2).

Again, patients with severe/necrotizing AP were younger than patients with mild AP, and SOFA, SAPS II, and APACHE II scores at ICU admission were unable to discriminate between the two groups (Table 2). The median length-of-stay on the ICU was significantly longer in patients with severe/necrotizing AP as compared to mild AP (13 vs. 4 days, *P* = 0.02).

**Table 2:** Clinical characteristics of patients with acute pancreatitis (ICU cohort)

Characteristics	Day 1 (Admission to ICU)		
	Severe or necrotizing (n = 33)	Non-severe edematous (n = 26)	*P* value
Age (years)	48 (38;61)	69 (49;77)	0.007
Male sex	23 (70%)	14 (54%)	0.22
BMI (kg/m.^2^)	27.0 (23.2;33.2)	27.3 (24.6;29.3)	0.91
Etiology			
Biliary Alcoholic Other	10 (30%) 11 (33%) 12 (36%)	7 (27%) 9 (35%) 10 (38%)	0.73
Hemoglobin (g/dl)	12.9 (10.5;15.1)	13.4 (10.9;15.8)	0.53
Hematocrit (%)	37 (31;42)	39 (33;47)	0.26
INR	1.1 (1.1;1.3)	1.1 (1.0;1.3)	0.67
PTT (s) Platelets (/nl)	26.3 (24.1;31.3) 213 (144;278)	29.0 (25.0;32.8) 213 (121;307)	0.39 0.67
Ca^2+^ (mmol/l)	1.99 (1.78;2.10)	2.09 (1.93;2.24)	0.80
LDH (U/l)	312 (219;605)	258 (201;405)	0.15
Bilirubin (mg/dl)	1.1 (0.7;2.6)	0.85 (0.4;2.6)	0.25
ALP (U/l)	85 (58;129)	100 (72;124)	0.53
GGT (U/l)	143 (77;304)	222 (79;493)	0.32
Amylase (U/l)	554 (299;888)	210 (93;1248)	0.23
Lipase (U/l)	506 (230;1509)	968 (545;3388)	0.13
ALT (U/l)	64 (28;138)	71 (29;231)	0.46
AST (U/l)	62 (31;128)	67 (38;250)	0.37
Albumin (g/l)	28 (26;31)	32 (27;37)	0.09
Glucose (mg/dl)	131 (102;181)	124 (94;149)	0.21
Creatinine (µmol/l)	80 (52;107)	106 (55;190)	0.15
SOFA score	5 (2.5;8.5)	5 (0.25;8)	0.56
SAPS II score	25 (19;37)	30 (20.5;39)	0.42
APACHE II score	16 (8;21)	16 (8;24)	0.90
Ranson 0 h (points)	1 (1;2)	1 (1;2)	0.96
CRP (mg/l)	142 (51;232)	67 (22;163)	0.06
PCT (ng/ml)	0.9 (0.3;8.6)	0.7 (0.2;3.0)	0.47
WBC (/nl)	13.3 (8.9;17.1)	12.2 (7.55;18.5)	0.69
sCD206 (mg/l)	1.76 (1.43;2.82)	1.25 (0.73;1.76)	0.005
vWF:Ag (%)	240 (186;464)	184 (140;308)	0.03
vWF:RCo (%)	177 (87;339)	129 (100;301)	0.67

*P* values from Mann–Whitney *U* test or Fisher’s exact test. Medians with first and third quartile or frequencies with percentages are shown

### Biomarkers of inflammation

In the prospective cohort, white blood cell count (14.6 vs. 11.9 /nl, *P* = 0.16), C-reactive protein (82.5 vs. 24.9 mg/l, *P* = 0.12), and procalcitonin (0.48 vs. 0.22 ng/ml, *P* = 0.10) did not significantly differ between patients with severe/necrotizing AP vs. non-severe edematous AP at hospital admission (Table 1, Additional file 1: Fig. S1).

In line with these results, white blood cell count (13.3 vs. 12.2 /nl, p = 0.69), C-reactive protein (142 vs. 67 mg/l, p = 0.06), and procalcitonin (0.9 vs. 0.7 ng/ml, *P* = 0.47) did not significantly differ between the both disease courses in the ICU cohort at ICU admission (Table 2, Additional file 1: Fig. S1).

**Fig. 1:**
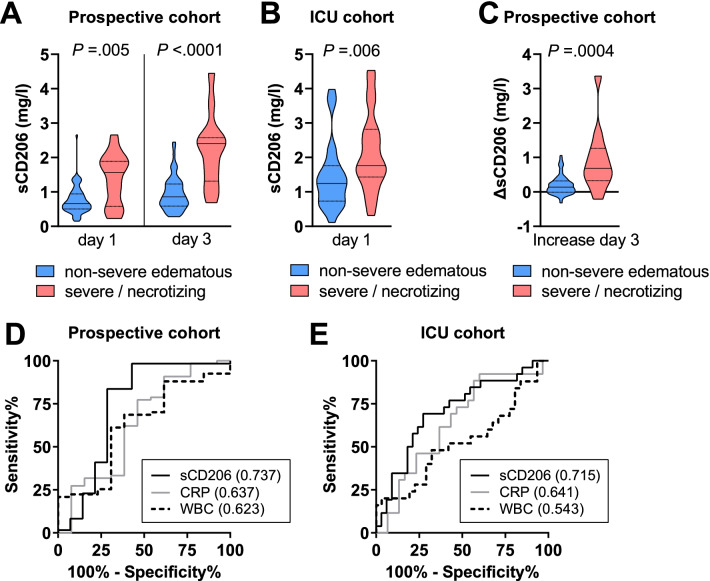
Serum concentrations of the soluble mannose receptor sCD206 in patients with acute pancreatitis. **A** sCD206 on admission to hospital (day 1) and after 48 h (day 3). **B** sCD206 on admission to the intensive care unit (ICU). **C** Differences in sCD206 between day 3 and day 1 (positive values indicate increases). Patients were stratified for the presence of organ failure or necrosis during follow-up. Violin plots with medians (solid) and quartiles (dotted) are shown. Day 1: hospital admission. *P* values from Mann–Whitney *U* test. **D**–**F** Receiver operating characteristics (ROC) curves for sCD206 predicting a non-severe edematous course, white blood cell count (WBC), and C-reactive protein (CRP) on admission for discrimination between patients who develop severe/necrotizing and patients with mild acute pancreatitis. **D** Prospective cohort, **E** ICU cohort

In contrast to conventional inflammation markers, the macrophage activation marker sCD206 was significantly elevated in patients who developed severe/necrotizing AP as compared to patients with non-severe edematous AP in both, the prospective cohort at hospital admission (1.57 vs. 0.66 mg/l, *P* = 0.005) and the ICU cohort at ICU admission (1.76 vs. 1.25 mg/l, *P* = 0.006) (Fig. 1A, B). The median increase in sCD206 from hospital admission to day 3 increase was higher in patients with severe/necrotizing AP (Fig. 1C), resulting in a significant higher sCD206 concentration in patients with severe/necrotizing AP at day 3 (Table 1).

Comparing the diagnostic accuracy of the inflammatory biomarkers CRP, WBC, and sCD206 at day 1 by ROC curve analysis confirmed the highest accuracy for sCD206 in the prospective cohort (AUROC 0.737; 95% CI 0.544–0.929) as well as in the ICU cohort (AUROC 0.715, 95% CI 0.575–0.854) (Fig. 1D, E).

Next, we investigated whether sCD206 concentrations would be sufficient to rule out the development of severe/necrotizing AP. Based on the maximum likelihood ratio (LR) in ROC curve analysis, sCD206 hospital admission levels of < 1.07 mg/l indicated a non-severe course of AP with moderate sensitivity (83%) and specificity (71%) in the prospective cohort (Likelihood ratio: 2.9) and sCD206 ICU admission levels of < 0.88 mg/l indicated a non-severe course of AP with low sensitivity (35%) and high specificity (91%) in the retrospective ICU cohort (Likelihood ratio: 3.8).

Consistent with inflammatory activation of the monocyte/macrophage compartment, the surface expression of the immune regulatory Mer tyrosine kinase (MERTK) on circulating CD14 + monocytes cells was increased as early as day 1 in patients who developed severe/necrotizing AP compared to patients who did not (Additional file 1: Fig. S2).

### Markers of endothelial function and hemostasis

Routine markers of hemostasis including INR, PTT, and platelets did not differ between patients with and without severe/necrotizing AP in both cohorts at hospital or ICU admission (Tables 1 and 2). D-Dimers (896 vs. 468 µg/l, *P* = 0.26) and fibrinogen (3.2 vs. 3.8 g/l, *P* = 0.50) were measured in the prospective cohort only and did not differ at presentation (Table 1). D-Dimer levels significantly differed between the two groups at day 3 (Additional file 1: Fig. S3).

**Fig. 2:**
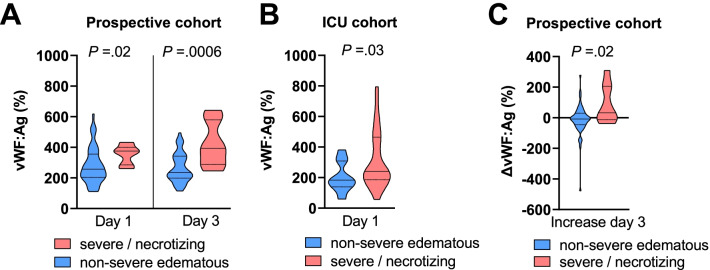
Von Willebrand Factor (vWF) antigen in patients with acute pancreatitis. **A** vWF:Ag on admission to hospital (day 1) and after 48 h (day 3). **B** vWF:Ag on admission to the intensive care unit (ICU). **C** Differences in vWF:Ag between day 3 and day 1 (positive values indicate increases). Patients were stratified for the presence of organ failure or necrosis during follow-up. Violin plots with medians (solid) and quartiles (dotted) are shown. Day 1: hospital admission (prospective cohort) or ICU admission (ICU admission). *P* values from Mann–Whitney *U* test

In both cohorts, vWF:Ag levels were significantly higher on hospital or ICU admission (median 375% vs. 257%, *P* = 0.02 in the prospective cohort; median 240% vs. 184%, *P* = 0.03 in the ICU cohort) in patients with severe/necrotizing AP (Fig. 2A, B). Patients who developed severe/necrotizing AP had a median absolute increase of vWF:Ag between day 1 and day 3 of 32% in contrast to patients with non-severe edematous pancreatitis, who experienced a median decrease of 8% (Fig. 2C).

vWF:Ag had moderate diagnostic accuracy in discriminating patients with severe/necrotizing AP from patients with non-severe edematous AP in the prospective cohort (AUROC 0.750; 95% CI 0.624–0.876) and in the ICU cohort (AUROC 0.718; 95% CI 0.569–0.866) on day 1, which was even higher at day 3 (AUROC 0.829; 95% CI 0.705–0.952).

vWF function as indicated by the vWF ristocetin cofactor activity (vWF:RCo) was significantly higher in patients with severe/necrotizing AP at day 1 (median 401% vs. 266%, *P* = 0.014) in the prospective cohort but not in the ICU cohort (median 177% vs. 129%, *P* = 0.67). The levels of the vWF-cleaving protease ADAMTS13 antigen (0.54 IU/ml vs. 0.57 IU/ml; *P* = 0.58) and activity (0.38 vs. 0.37 IU/ml, p = 0.80) did not differ between patients with severe/necrotizing AP and mild AP.

### sCD206 and vWF as predictors of a severe or necrotizing course of AP

We used binary logistic regression models to evaluate the ability of sCD206 and VWF antigen to predict the occurrence of severe or necrotizing pancreatitis. The univariate odds ratios for severe/necrotizing AP were 6.08 (1.77–20.81) and 2.90 (1.19–7.05) per 1-log_e_[mg/l] increase in sCD206 and 9.33 (1.05–82.81) and 3.34 (1.04–10.67) per 1-log_e_[%] increase in vWF:Ag, in the prospective cohort and in the ICU cohort, respectively. To address potential confounders, binary logistic regression analysis after adjustment for the etiology of AP (stratified by biliary vs. alcoholic vs. other) was performed. In multivariable models, sCD206 and vWF remained independent predictors of severe or necrotizing pancreatitis after adjustment for etiology and age when used as a continuous variable (Table 3). When dichotomized using the highest quartiles (Q4) as the cutoff, sCD206 but not VWF antigen remained a significant indicator in both cohorts in univariate and in multivariable models. Given the intercorrelation of sCD206 and vWF:RCo (Spearman's rho = 0.496 in the prospective cohort; P < 0.01), both variables were not used together in a multivariable logistic regression model.

**Table 3:** Binary logistic regression analysis to identify patients with severe/necrotizing pancreatitis

Characteristics	Univariate analysis		Adjusted for etiology^#^		Adjusted for etiology^#^ and age	
	Univariate odds ratio (95% CI)	*P* value	Adjusted odds ratio (95% CI)	*P* value	Adjusted odds ratio (95% CI)	*P* value
sCD206*						
Prospective cohort ICU cohort	6.08 (1.77–20.81) 2.90 (1.19–7.05)	0.004 0.02	4.29 (1.20–15.37) 2.95 (1.21–7.22)	0.03 0.02	5.96 (1.46–24.30) 3.33 (1.22–9.13)	0.01 0.02
sCD206 Q4^$^						
Prospective cohort ICU cohort	9.18 (2.54–33.23) 4.38 (1.08–17.71)	0.001 0.04	8.69 (1.90–39.70) 4.45 (1.09–18.11)	0.005 0.04	17.54 (2.63–117.1) 8.31 (1.66–41.68)	0.003 0.01
vWF:Ag*						
Prospective cohort ICU cohort	9.33 (1.05–82.81) 3.34 (1.04–10.67)	0.04 0.04	102.0 (2.1–5016.8) 3.69 (1.11–12.27)	0.02 0.03	205.0 (2–2–18,729.5) 8.40 (1.61–43.92)	0.02 0.01
vWF:Ag Q4^$^						
Prospective cohort ICU cohort	5.25 (1.19–23.17) 4.00 (0.77–20.92)	0.03 0.10	9.45 (0.90–98.92) 4.02 (0.74–21.71)	0.06 0.11	12.43 (0.96–161.76) 8.69 (1.18–64.13)	0.05 0.03

*log_e_-transformed values were used. ^#^stratified by biliary vs. alcoholic vs. other etiologies. ^$^The fourth quartile in each cohort was compared to the other quartiles (Q1–Q3) within the cohort

### sCD206 and vWF in patients with bacterial infections

**Fig. 3:**
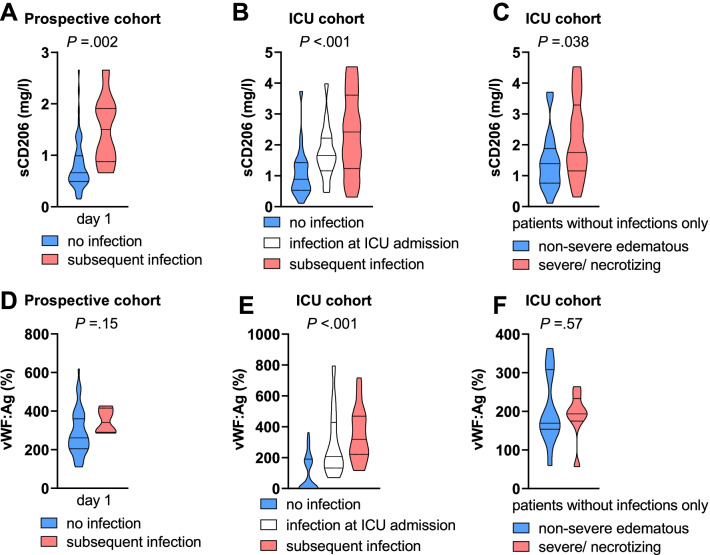
Association between infections and soluble mannose receptor sCD206 and von Willebrand factor (vWF) antigen concentrations. **A** sCD206 concentrations in serum and **D** VWF antigen in plasma at hospital admission (day 1) in patients stratified by subsequent occurrence of infection. No patient in the prospective cohort had a manifest bacterial infection on admission. **B** sCD206 and E) vWF antigen on admission to the intensive care unit (ICU) stratified by the presence of manifest infection and the subsequent occurrence of infection. Subgroup analysis of patients admitted to the ICU without manifest infection with **C** serum sCD206 levels and **F** plasma vWF antigen stratified by the presence of organ failure or necrosis during follow-up. Violin plots with medians (solid) and quartiles (dotted) are shown. P values from Mann–Whitney *U* test or Kruskal–Wallis test

Because circulating sCD206 levels are elevated in patients with impaired intestinal barrier function^33^ and in patients with manifest bacterial or fungal infections^34^, we examined the presence of bacterial infections as a confounding factor in our analysis. In the prospective cohort, no patients were diagnosed with bacterial or fungal infection on hospital admission, and eight patients developed infection during follow-up. In the retrospective ICU cohort, 15 patients had a proven or suspected bacterial infection on admission, and an additional 21 patients developed an infection during follow-up (Additional file 1: Table S2). Patients who did not develop infection had lower serum sCD206 concentrations than patients who presented with or later developed infection (Fig. 3A, B). However, when only patients without infections at ICU admission were considered for analysis, sCD206 still differed significantly between patients with mild AP and patients with severe/necrotizing AP (Fig. 3C). The association between vWF:Ag and manifest or subsequent infections was less consistent (Fig. 3D–F).

## Discussion

In this study, we report that soluble mannose receptor CD206, a biomarker of macrophage activation, and von Willebrand factor antigen, a biomarker of endothelial perturbation, significantly differ between patients with severe or necrotizing AP and patients with mild AP on the day of hospital admission, 48 h later, and at ICU admission. In contrast, prognostic scores of AP severity and organ failure were not able to identify patients with a more severe disease course in these cohorts at hospital or ICU admission.

Predicting the course of AP on admission is challenging, as necrosis has not yet developed and organ failure may be absent or occur in a transient manner. In addition, early markers of organ dysfunction may be influenced by systemic inflammation^35^. An APACHE II Score above 8 or elevated serum creatinine levels > 24 h after admission are associated with persistent organ failure in acute pancreatitis^36^ but there remains a gap of 24 h between admission and the estimation of prognosis. Finding satisfying markers for this time period of early and decisive clinical decisions has been a struggle and no definitive marker has been established.

Potential early predictors for the course of pancreatitis are cellular markers of inflammation. The neutrophil-to-lymphocyte ratio has been described to identify patients with organ failure or severe pancreatitis in a recent study^37^. Given the pivotal role of immune cells of the myeloid lineage in severe AP, we investigated sCD206 as a marker of macrophage activation shed into the circulation^38,39^. Markers of macrophage activation such as sCD206 are increased in patients with fungal infections^16,34,40^, pneumonia^41^, sepsis^42^ and liver disease^43,44^, indicate poor prognosis in alcoholic hepatitis^43^ and acute-on-chronic liver failure^45^ and have the potential to improve established scoring systems. Analysis of circulating monocytes showed phenotypic alterations in the myeloid compartment, such as increased surface expression of the Mer tyrosine kinase in patients that will develop a complicated course of AP, already present on admission.

Although the extent of pancreatic tissue injury is a likely explanation for the release of sCD206 into the circulation, we cannot exclude a concurrent microbial infection in this situation. Our analysis shows that elevated levels of sCD206 were observed in both, in patients with concurrent infections admitted to the intensive care unit and in patients who developed infections at later timepoints. However, even after excluding patients with manifest infections at blood collection, sCD206 concentrations were significantly associated with severe disease progression in both cohorts.

Previous studies^46^ have demonstrated increased intestinal permeability in the early phase of AP before the development of bacteremia and organ failure, which may influence sCD206 levels before the onset of manifest infection^33^. As with other inflammatory markers, sCD206 concentration must be evaluated in the context of the likelihood of sterile inflammation vs. bacterial or fungal infection^34^.

Given the role of microcirculatory dysfunction in AP and the close relationship of inflammatory and hemostatic systems^21–23^, another potential target for early identification of patients with severe or necrotizing pancreatitis are markers of endothelial perturbation and hemostasis. Although the vast majority of patients with AP presented elevated vWF:Ag levels as previously described^24–27^, a more severe course was associated with significantly higher vWF:Ag already on admission to hospital or the ICU. As an acute-phase-protein and procoagulant glycoprotein, vWF is secreted in multimeric formulation by activated endothelial cells^47,48^. Our observations are in line with previous studies, linking higher vWF to pancreatic necrosis^49^ and respiratory failure^50^. The key regulator of vWF multimer size, which is also crucial for vWF activity, is its protease ADAMTS13. Interestingly, we did not observe a differential regulation of ADAMTS13 with respect to AP severity despite its modulation by inflammation, infections and organ failure^51–58^ and the inverse correlation of ADAMTS13 and the APACHE II score in patients with severe pancreatitis, that had been reported in previous publications^59^.

This study has several limitations, which have to be taken into account. First of all, we cannot rule out that the observed changes in the immune system and in inflammatory and coagulation parameters are influenced by other aspects connected to or independent from pancreatitis. This risk was minimized by excluding patients with comorbidities, that have been known to influence vWF activity. We further tried to eliminate potential confounders, such as etiology by performing multivariate analyses. Second, the duration of symptoms prior to hospital admission was not assessed in our study. Minimizing such confounding bias especially in ICU patients is difficult but was dealt with by excluding patients, whose index day of symptoms was more than 24 h before ICU admission. Third, the AP case fatality is low, which does not allow the investigation of harder end points, such as mortality, in adequately powered studies. In addition, overall mortality of AP has been declining^60^.

One potential limitation is the dichotomization of patients used, which differs from current classification systems with three (Revised Atlanta classification ^2^) or four (Determinant-Based Classification ^61^) severity strata. This not only improves statistical power by reducing the number of severities, but is also consistent with current clinical concepts^62^. Patients with pancreatic necrosis were grouped together with patients with severe AP, because local complications may often require a variety of interventions to avoid a fatal outcome. Patients with moderately severe AP and transient organ failure were grouped together with mild AP, because transient organ failure is associated with a generally good prognosis, significantly less local complications ^63^, and no need for transfer to a tertiary medical center or an ICU ^64^.

## Conclusions

Our findings indicate that surrogates of macrophage activation and endothelial function are promising early biomarkers to identify patients at risk for a complicated course of AP. In particular, we herein report that sCD206 was a better biomarker than routine inflammatory parameters to identify patients at risk of complicated courses of AP at hospital admission or ICU admission. Further prospective studies are needed to investigate whether the inclusion of early or repeated measurements in the existing scoring system proves effective in better identifying patients at risk of severe AP and whether it can be used for better risk stratification in the future.

## Supplementary Information

**Additional file 1. **Additional methods. **Table S1.** Clinical course and outcome of patients with acute pancreatitis (prospective cohort). **Table S2.** Clinical course and outcome of patients with acute pancreatitis (ICU cohort). **Fig. S1.** White blood cell count, C-reactive protein and procalcitonin in patients with acute pancreatitis. **Fig. S2.** Surface expression of the MHC class II molecule HLA-DR and the Mer tyrosine kinase (MERTK) on circulating CD14+ cells. **Fig. S3. **Fibrinogen and D-Dimer concentrations on admission in patients with acute pancreatitis.

## Data Availability

The data sets used and/or analysed during the current study are available from the corresponding author on reasonable request.

## Abbreviations

ADAMTS13: A disintegrin and metalloproteinase with a thrombospondin type 1 motif, member 13
AP: Acute pancreatitis
APACHE: Acute Physiology and Chronic Health Evaluation
ALT: Alanine aminotransferase
AST: Aspartate aminotransferase
BISAP: Bedside index for severity in acute pancreatitis
CI: Confidence interval
CRP: C-reactive protein
ELISA: Enzyme-linked immunosorbent assay
GGT: Gamma-glutamyl transferase
INR: International normalized ratio
IQR: Interquartile range
PCT: Procalcitonin
RCo: Ristocetin cofactor
ROC: Receiver operating characteristic
SIRS: Systemic inflammatory response syndrome
SOFA: Sequential Organ Failure Assessment
vWF: Von Willebrand factor
WBC: White blood cells
