# Supplementary material for: Soluble mannose receptor CD206 and von Willebrand factor are early biomarkers to identify patients at risk for severe or necrotizing acute pancreatitis
Source: J Intensive Care. 2022 Jun 11;10:28. doi: 10.1186/s40560-022-00619-2 (PMC9188125; doi:10.1186/s40560-022-00619-2)
Supplement: Supplementary file 1 — Additional file 1. Additional methods. Table S1. Clinical course and outcome of patients with acute pancreatitis (prospective cohort). Table S2. Clinical course and outcome of patients with acute pancreatitis (ICU cohort). Fig. S1. White blood cell count, C-reactive protein and procalcitonin in patients with acute pancreatitis. Fig. S2. Surface expression of the MHC class II molecule HLA-DR and the Mer tyrosine kinase (MERTK) on circulating CD14+ cells. Fig. S3. Fibrinogen and D-Dimer concentrations on admission in patients with acute pancreatitis. [file 40560_2022_619_MOESM1_ESM.docx]

Additional file 1 to the manuscript

**Soluble mannose receptor CD206 and von Willebrand Factor are early biomarkers to identify patients at risk for severe or necrotizing acute pancreatitixqs**

**Authors:**Philipp A Reuken^1*^ [philipp.reuken@med.uni-jena.de](mailto:philipp.reuken@med.uni-jena.de)
Jonathan F Brozat^2*^ jbrozat@ukaachen.de
Stefanie Quickert^1^ [stefanie.quickert@med.uni-jena.de](mailto:stefanie.quickert@med.uni-jena.de)
Oluwatomi Ibidapo-obe ^2^ [oibidapo@ukaachen.de](mailto:oibidapo@ukaachen.de)
Johanna Reißing^2^ [joreissing@ukaachen.de](mailto:jreissing@ukaachen.de)
Anika Franz^1^ [Anika.franz@med.uni-jena.de](mailto:sven.stengel@med.uni-jena.de)
Sven Stengel^1^ [sven.stengel@med.uni-jena.de](mailto:sven.stengel@med.uni-jena.de)
Ulf K-M Teichgräber^3^ [ulf.teichgraeber@med.uni-jena.de](mailto:ulf.teichgraeber@med.uni-jena.de)
Michael Kiehntopf^4^ [michael.kiehntopf@med.uni-jena.de](mailto:michael.kiehntopf@med.uni-jena.de)
Christian Trautwein^2^ [ctrautwein@ukaachen.de](mailto:ctrautwein@ukaachen.de)
Andreas Stallmach^1^ [andreas.stallmach@med.uni-jena.de](mailto:andreas.stallmach@med.uni-jena.de)
Alexander Koch^2#^ [akoch@ukaachen.de](mailto:akoch@ukaachen.de)
Tony Bruns^1.2#^ [tbruns@ukaachen.de](mailto:tbruns@ukaachen.de)

*PAR and JFB are equally contributing first authors.
^#^TB and AK are equally contributing senior authors.

**Affiliations:**^1^ Department of Internal Medicine IV, Jena University Hospital, Friedrich Schiller University Jena, Germany
^2^ Department of Internal Medicine III, University Hospital RWTH Aachen, Aachen, Germany.
^3^ Department of Radiology, Jena University Hospital, Friedrich Schiller University Jena, Germany
^4^ Department of Clinical Chemistry and Laboratory Diagnostics, Jena University Hospital, Jena, Germany..

Additional file 1 **methods:**

*Flow cytometry*

Mononuclear cells were isolated from EDTA blood using Lympholyte-H separation media (Cedarlane, Burlington, Ontario, Canada) and washed in phosphate buffered saline (PBS) (Thermo Fisher Scientific, Waltham, MA, USA). For the analysis of surface antigens, cells were stained with LIVE/DEAD™ Fixable Aqua Dead Cell Stain Kit (Thermo Fisher Scientific) and incubated with fluorochrome-conjugated primary Abs at optimal dilutions at 4°C in PBS containing 2% fetal calf serum (FCS) and 2mM EDTA. Cells were stained with the following monoclonal antibodies against surface markers: CD14 (clone TÜK4), HLA-DR (clone G46-6), and MERTK (clone 125518), which were purchased from BD Biosciences (Heidelberg, Germany), R&D Systems (Wiesbaden, Germany) or from Miltenyi Biotec (Bergisch Gladbach, Germany). After staining, cells were rinsed twice, re-suspended in PBS containing 2% FCS and 2mM EDTA and analyzed in a Beckman Coulter CytoFLEX flow cytometer. Single color staining, isotype-matched controls (IMC) and fluorescence minus one (FMO) controls were performed.

| Additional file 1 **Table S1.** Clinical course and outcome of patients with acute pancreatitis (prospective cohort) | | | |
| --- | --- | --- | --- |
| Characteristics | Severe/necrotizing  (n=14) | Mild  (n=67) | *P* value |
| Length of hospital stay (days) | 20 (14; 29) | 8 (5; 14) | <0.001 |
| Admission to ICU/IMC (n) | 9 (64.3%) | 12 (15.4%) | 0.001 |
| Organ failure | 4 (28.6%) | 0 |  |
| Transient organ failure <48h | 0 | 11 (16.4 %) |  |
| Time to diagnosis of necrosis | 7 (6; 10) | N/A |  |
| Infected necrosis* | 4 (28.6%) | N/A |  |
| Drainage/necrosectomy | 3 (21.4%) | N/A |  |
| In-Hospital-Survival | 13 (93%) | 67 (100%) | 0.17 |

**Streptococcus mitis*, *Enterococcus faecalis*, *Klebsiella pneumoniae,* coagulase-negative *Staphylococcus* spp.

| Additional file 1 **Table S2**. Clinical course and outcome of patients with acute pancreatitis (ICU cohort) | | |
| --- | --- | --- |
|  | Severe/necrotizing  (n=33) | Mild  (n=26) |
| Primary reason for ICU admission  Alcohol withdrawal symptoms  Bleeding  Bradycardia/Trachycardia  Impacted concrement  Organ failure  Pain management  Short-term monitoring | 5  1  1  0  24  1  1 | 5  0  2  5  7*  1  6 |
| Length of ICU stay (days) | 12 (4; 28) | 3 (2; 5) |
| Infection at ICU admission  Cholangitis  Respiratory infection  Urinary tract infection  Skin and soft tissue infection | 0  7  1  0 | 3  3  0  1 |
| Infection after ICU admission^#^  Infected pancreatic necrosis  Blood stream infection  Cholangitis  *C. difficile* infection  Respiratory infection  Urinary tract infection  Skin and soft tissue infection  Other | 14  9  0  3  15  5  1  3 | 0  0  0  0  5  1  0  0 |
| Time to infection (days) | 7 (0; 13) | 2 (0; 6) |
| In-Hospital mortality | 4 (12%) | 0 |

*all with transient organ failure

^#^sum exceeds 100% due to multiple infections

For continuous data medians with first and third quartiles are shown.

Additional file 1  **Figure S1.**

**White blood cell count, C-reactive protein and procalcitonin in patients with acute pancreatitis.** Patients were stratified for failure or necrosis according to the revised Atlanta classification. Violin plots with medians (solid) and quartiles (dotted) are shown. Day 1: hospital admission. P values from Mann-Whitney U test.

Additional file 1  **Figure S2.**

**Surface expression of the MHC class II molecule HLA-DR and the Mer tyrosine kinase (MERTK) on circulating CD14+ cells.** Patients from the prospective cohort (n=34) were stratified for failure or necrosis according to the revised Atlanta classification. Violin plots with medians (solid) and quartiles (dotted) of median fluorescence intensities (MFI) normalized to fluorescence minus one (FMO) controls are shown. P values from Mann-Whitney U test.

Additional file 1 **Figure S3**

**Fibrinogen and D-Dimer concentrations on admission in patients with acute pancreatitis.** Patients were stratified for failure or necrosis according to the revised Atlanta classification. Violin plots with medians (solid) and quartiles (dotted) are shown. P values from Mann-Whitney U test.
